# Supplementary material for: Deep learning for pediatric chest x-ray diagnosis: Repurposing a commercial tool developed for adults
Source: PLoS One. 2025 Jul 24;20(7):e0328295. doi: 10.1371/journal.pone.0328295 (PMC12289065; doi:10.1371/journal.pone.0328295)
Supplement: S1 Table — (DOCX) [file pone.0328295.s001.docx]

**S1 Table: Results of the algorithm performance in children aged 2-6 years.**

| **Pathology** | **Cases (n=509)** | **AUC (95% CI)** | | **Accuracy (95% CI)** | | **Sensitivity** | | **Specificity** | | **PPV** | | **NPV** | |  |
| --- | --- | --- | --- | --- | --- | --- | --- | --- | --- | --- | --- | --- | --- | --- |
| **Relevant pathology** | 143 (27.3%) | | 0.91 (0.88-0.94) | | 78 (74.1-81.5) | | 88.1 (126/143) | | 74.0 (271/366) | | 57.0 (126/221) | | 94.1 (271/288) | |
| **Pleuroparenchymal pathology** | 116 (22.8%) | | 0.90 (0.87-0.94) | | 79.2 (75.4-82.6) | | 86.2 (100/116) | | 77.1 (303/393) | | 52.6 (100/190) | | 95.0 (303/319) | |
| **Mediastinal pathology** | 58 (11.4%) | | 0.91 (0.88-0.95) | | 85.5 (82.1-88.4) | | 70.7 (41/58) | | 87.4 (394/451) | | 41.8 (41/98) | | 95.9 (394/411) | |
| **Consolidation** | 108 (21.2%) | 0.88 (0.85-0.92) | | 78.6 (74.8-82.1) | | 82.4 (89/108) | | 77.5 (311/401) | | 49.7 (89/179) | | 94.2 (311/330) | |  |
| **Atelectasis** | 14 (2.8%) | 0.97 (0.94 - 1) | | 98 (96.4-99.1) | | 64.3 (9/14) | | 99.0 (490/495) | | 64.3 (9/14) | | 99.0 (490/495) | |  |
| **Nodule** | 7 (1.4%) | 0.91 (0.74 - 1) | | 93.1 (90.6-95.2) | | 85.7 (6/7) | | 93.2 (468/502) | | 15.0 (6/40) | | 99.8 (468/469) | |  |
| **Pleural Effusion** | 11 (2.2%) | 0.96 (0.94-0.99) | | 98.0 (95.9-98.8) | | 45.4 (5/11) | | 98.8 (492/498) | | 45.4 (5/11) | | 98.8 (492/498) | |  |
| **Pneumothorax** | 0 (0%) | NA | | 100 (99.3-100) | | NA | | 100 | | NA | | NA | |  |
| **Cardiomegaly** | 54 (10.6%) | 0.93 (0.90-0.96) | | 87.2 (84-90) | | 72.2 (39/54) | | 89.0 (405/455) | | 43.8 (39/89) | | 96.4 (405/420) | |  |
| **Mediastinal widening** | 5 (1%) | 0.81 (0.61-1) | | 94.7 (92.4-96.5) | | 40.0 (2/5) | | 95.2 (480/504) | | 7.7 (2/26) | | 99.4 (480/483) | |  |

AUC= Area under the receiver operating characteristic curve

PPV= Positive Predictive Value

NPV= Negative Predictive Value
