# Supplementary material for: Deep learning for pediatric chest x-ray diagnosis: Repurposing a commercial tool developed for adults
Source: PLoS One. 2025 Jul 24;20(7):e0328295. doi: 10.1371/journal.pone.0328295 (PMC12289065; doi:10.1371/journal.pone.0328295)
Supplement: S2 Table — (DOCX) [file pone.0328295.s002.docx]

**S2 Table: Results of the algorithm performance in children aged 7-14 years**

| **Pathology** | **Cases (n=449)** | **AUC (95% CI)** | **p-value** | **Accuracy (95% CI)** | **Sensitivity** | **Specificity** | **PPV** | **NPV** |
| --- | --- | --- | --- | --- | --- | --- | --- | --- |
| **Relevant pathology** | **58 (13%)** | **0.96 (0.95-0.98)** | **<0.001** | **89.7 (86.5-92.4)** | **86.2 (50/58)** | **90.3 (353/391)** | **56.8 (50/88)** | **97.8 (353/361)** |
| **Pleuroparenchymal pathology** | **46 (10.2%)** | **0.98 (0.97-0.99)** | **<0.001** | **92.2 (89.3-94.5)** | **91.3 (42/46)** | **92.3 (372/403)** | **57.5 (42/73)** | **98.9 (372/376)** |
| **Mediastinal pathology** | **21 (4.7%)** | **0.97 (0.95-0.99)** | **0.007** | **93.5 (90.1-95.6)** | **81 (17/21)** | **94.1 (403/428)** | **40.5 (17/42)** | **99 (403/407)** |
| **Consolidation** | **36 (8%)** | **0.98 (0.97-0.99)** | **<0.001** | **92.9 (90.1-95.1)** | **91.7 (33/36)** | **93 (384/413)** | **53.2 (33/62)** | **99.2 (384/387)** |
| Atelectasis | 14 (3.1%) | 0.96 (0.93-0.99) | 0.60 | 96.4 (94.3-98.0) | 35.7 (5/14) | 98.4 (428/435) | 41.7 (5/12) | 97.9 (428/437) |
| Nodule | 2 (0.4%) | 0.99 (0.99-1) | 0.31 | 94.0 (91.4-96.0) | 100 (2/2) | 93.9 (420/447) | 06.9 (2/29) | 100 (420/420) |
| **Pleural Effusion** | **16 (3.6%)** | **0.99 (0.98-0.99)** | **0.04** | **97.5 (95.6-98.8)** | **81.3 (13/16)** | **98.1 (425/433)** | **61.9 (13/21)** | **99.3 (425/428)** |
| **Pneumothorax** | **2 (0.4%)** | **1.00 (1-1)** | **NA** | **99.8 (98.8-100)** | **100 (2/2)** | **99.8 (446/447)** | **66.7 (2/3)** | **100 (446/446)** |
| **Cardiomegaly** | **20 (4.4%)** | **0.97 (0.95-0.99)** | **0.02** | **94.2 (91.6-96.2)** | **80.0 (16/20)** | **94.9 (407/429)** | **42.1 (16/38)** | **99.0 (407/411)** |
| **Mediastinal widening** | **1 (0.2%)** | **0.98 (NA-NA)** | **NA** | **97.5 (95.6-98.8)** | **100 (1/1)** | **97.5 (437/448)** | **08.3 (1/12)** | **100 (437/437)** |

AUC= Area under the receiver operating characteristic curve

PPV= Positive Predictive Value

NPV= Negative Predictive Value

Bold text indicates changes compared to 2-6 years.
