# Supplementary figures and images for: Deep learning for pediatric chest x-ray diagnosis: Repurposing a commercial tool developed for adults
Source: PLoS One. 2025 Jul 24;20(7):e0328295. doi: 10.1371/journal.pone.0328295 (PMC12289065; doi:10.1371/journal.pone.0328295)

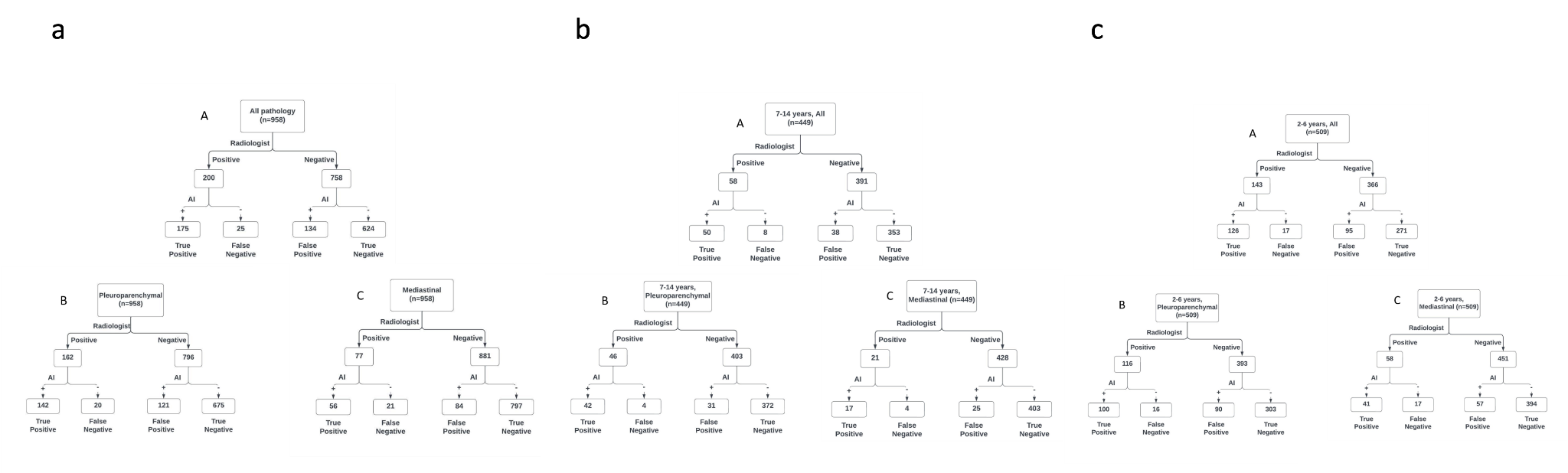

Supplement: S1 Fig — A) All relevant pathologies. B) Pleuroparenchymal pathologies. C) Mediastinal pathologies. (TIF) [file pone.0328295.s003.tif]

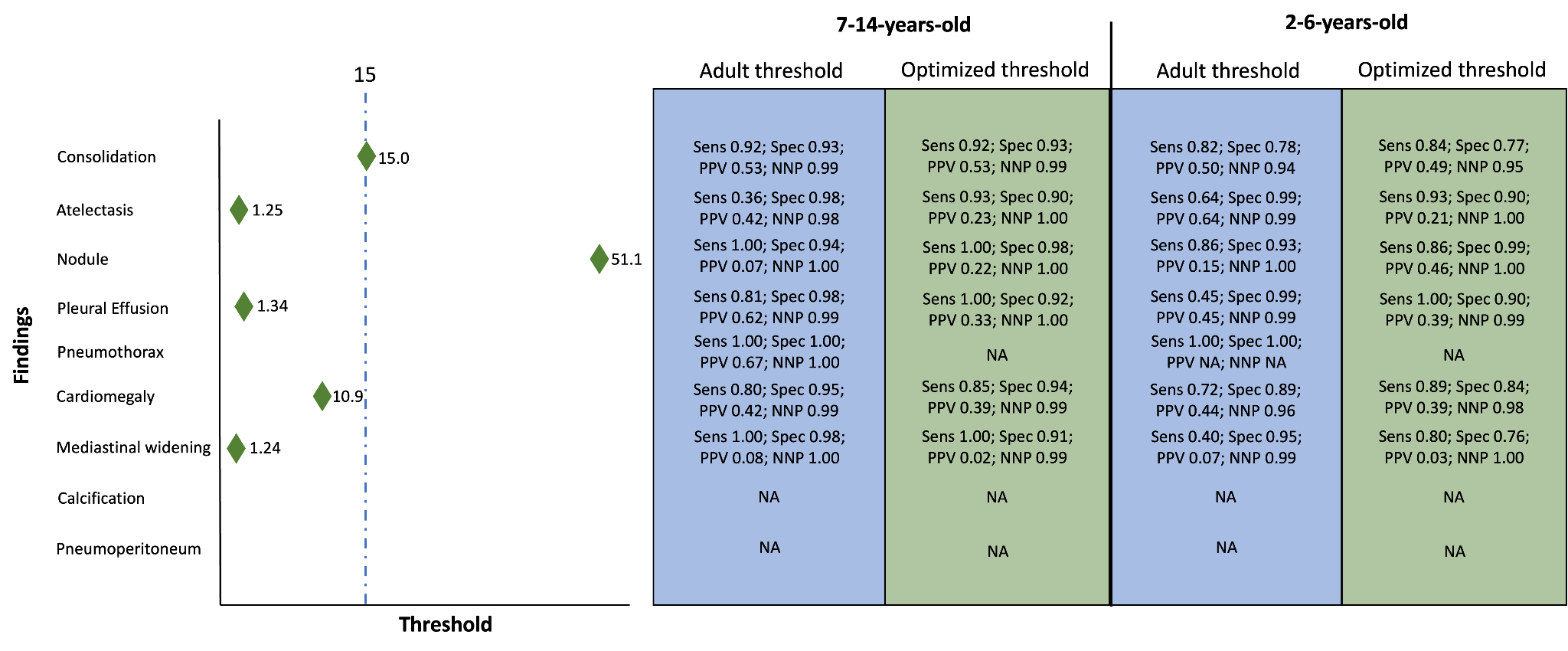

Supplement: S2 Fig — The dotted blue line represents the pre-defined vendor recommended threshold of 15, which is based on the optimal threshold identified for adults to dichotomize the continuous AI-output (0–100). The green diamonds show optimized cut-offs calculated for maximizing the sum of sensitivity and specificity. The performance metrics based on adult threshold of 15 (blue) and optimized cutoffs for children (green) are shown in the column on the right side (sens = sensitivity, spec = specificity, PPV = positive predictive value, NPV = negative predictive value) (TIF) [file pone.0328295.s004.tif]
