# Supplementary material for: Deep learning for pediatric chest x-ray diagnosis: Repurposing a commercial tool developed for adults
Source: PLoS One. 2025 Jul 24;20(7):e0328295. doi: 10.1371/journal.pone.0328295 (PMC12289065; doi:10.1371/journal.pone.0328295)
Supplement: Attachment — Submitted filename: CXR3_point-to-point_withresponse.docx [file pone.0328295.s006.docx]

# Point-by-Point Response to Reviewer Comments

PONE-D-25-04141

Deep Learning for Pediatric Chest Radiograph Diagnosis: Repurposing a Commercial Tool Developed for Adults

***General Response****: We thank the editor and reviewers for their thoughtful comments and have addressed all concerns in the revised version of the manuscript. We have also formatted the manuscript to suit the journal´s style and reorganized the layout to include the tables and figure texts in the manuscript. A point-by-point response to reviewers is provided below. The page and line numbers refer to the manuscript version with tracked changes.*

**Response to Reviewer 1**

We thank the reviewer for the critical evaluation of our work and are grateful for their very helpful comments and suggestions that we have incorporated in our revised manuscript as detailed below:

**Comment 1:**

I recommend naming the commercial tool directly in the abstract so that researchers interested in this tool can identify your paper more quickly.

**Response 1:**

We have added the name of the tool to the abstract. (Page: 3, Line: 5)

**Comment 2:**

Would mention out of the 958 how many had abnormal CXR (n=200) for a bit more context on the study population.

**Response 2:**

We thank the reviewer for this suggestion. We have added the following sentence to the **Abstract** to provide this context (Page: 3, Line: 17):

“*200 radiographs [20.9%] demonstrated at least one relevant pathology*”.

**Comment 3:**

The diagnostic performance of the AI-tool was validated using standard measures of accuracy using recommended and optimized thresholds to dichotomize the continuous AI output (0-100)” – clarify that the recommended thresholds are for adults.

**Response 3:**
The sentence was changed as follows for clarity (Page: 3, Lines: 12-14 ):

*“For this, the continuous AI output (ranging from 0-100) was binarized using vendor recommended thresholds recommended for adults and optimized thresholds identified for children.”*

**Comment 4:**

I am not a proponent of reporting on the accuracy of an algorithm. Accuracy can be highly misleading in imbalanced datasets as is the case with only 200 positives. For overall performance, I would report the AUC and then the sensitivity/specificity, specifying that this is against the adult-recommended threshold. For the age-stratified results, I would only report the AUCs in the abstract.

**Response 4:**

Thank you for your valuable feedback.

We have changed the abstract as follows (Page: 3, Lines: 17-23)

*“Using the adult threshold, the AI-tool showed a high performance for all relevant findings with an AUC 0.94 (95% CI: 0.92-0.95) and. In stratified analysis by age (2-7 vs. 7-14-years-old) a significantly higher performance (p<0.001) was found for older children with an AUC of 0.96 (95% CI: 0.94-0.98) with a sensitivity and specificity of 87.5% and 82.3% respectively, which further increased using optimized thresholds for children.”*

**Comment 5:**

Again, would name the actual CXR device here (in introduction).

**Response 5:**

We have made the following changes to the introduction: Page: 5, Line: 2

*“Here, we investigated the diagnostic performance of a commercially available AI-tool developed for adult chest radiograph interpretation (Lunit INSIGHT CXR) in a real-world clinical dataset of children aged 2-14 years old.”*

**Comment 6:**

The initials of the radiologists are still “XX”

**Response 6:**

The initials were changed to “PA” (Page: 7, Line: 12)

**Comment 7:**

Inconsistent nomenclature for figure (“Fig.” vs “Figures”)

**Response 7:**

We changed “Fig.” and “Figures” to “Fig” to comply with the journal´s formatting guidelines.

**Comment 8:**

“The following ten findings are detected by the algorithm: consolidation, atelectasis, nodule, fibrosis, calcification, pleural effusion, pneumothorax, pneumoperitoneum, cardiomegaly and mediastinal widening.” – could you provide the N for each of these that were used when training the model on the adult population. Would allow for a comparison with the pediatric cohort.

**Response 8:**
We would like to thank the reviewer for this question. We have contacted Lunit for this question and have included their response here:

“While we are unable to disclose the exact number of annotated training cases for each individual finding due to proprietary limitations, I can confirm that Lunit INSIGHT CXR was trained on a dataset of approximately 280,000 chest X-rays with annotation and the algorithm was specifically trained to detect the following ten key findings: Consolidation, atelectasis, nodule, fibrosis, calcification, pleural effusion, pneumothorax, pneumoperitoneum, cardiomegaly, and mediastinal widening. All images used for training were confirmed by one of the following methods: Original radiology report + pathology confirmation or radiology report + CT confirmation, or independent image review by at least one expert radiologist. This approach ensured high-quality ground truth labeling for accurate model training. The dataset consisted of PA and AP images from adult patients, with data sourced from multiple countries and acquired using equipment from 24+ X-ray device manufacturers, providing significant diversity across pathologies and imaging conditions.”

**Comment 9:**

You are missing the diagnostic performance metrics (AUC, sens, spec…) of the CAD in the adult population. I see it in figure 5, but this could be missed and should be in the main text to allow for comparison with what is achieved in pediatrics.

**Response 9:**

We thank the reviewer for this comment. We would like to clarify that the diagnostic performance metrics shown in Figure 5 reflect the performance of the AI tool in the pediatric population, using the recommended threshold of 15, which is based on the optimal threshold identified for adults. To improve clarity, we have now explicitly stated this in the main text and the figure. We would like to highlight that performance metrics for the adult population were not the focus of this analysis.

Page 14, Lines: 12 -23

*“The vendor-recommended threshold of 15 to binarize the continuous AI output (ranging from 0-100) is based on the optimal threshold identified for adults. Therefore, as a final step in our analysis, we calculated optimized cut-offs for children for the entire cohort. Overall, the optimal cut-off for the pediatric age group was found to be 44.5. This adjusted threshold yielded a similar AUC of 0.93 with an accuracy of 89.8% (95% CI: 87.7–91.6%), sensitivity of 80.0%, and specificity of 92.4%. Compared to the adult threshold, the pediatric-specific cut-off led to improvement in the specificity and the PPV, supporting a safer AI interpretation in children with a slight trade-off in the sensitivity at 80%. The sub-group specific thresholds for pleuroparenchymal and mediastinal subgroups are shown in the Fig 4 and for each pathology are shown in* ***S1 Fig.***

Page 14, Lines: 25-32

“***Fig 5:*** *Definition of optimal thresholds for the performance of the AI-tool in children. The dotted blue line represents the pre-defined vendor recommended threshold of 15, which is based on the optimal threshold identified for adults to dichotomize the continuous AI-output (0-100). The green diamonds show optimized cut-offs calculated for maximizing the sum of sensitivity and specificity for pleuroparenchymal and mediastinal subgroups in the entire cohort. The performance metrics based on adult threshold of 15 (blue) and optimized cutoffs for children (green) are shown in the column on the right side.”*

**Comment 10:**

When discussing threshold-based metrics (accuracy, sens, spec, PPV, NPV) it is good to remind the reader that this is benchmarked against the adult threshold.

**Response 10:**

We thank the reviewer for this suggestion. Accordingly, we added the following sentence to Statistical analysis:

Page: 9, Lines 23-24

“In addition, sensitivity, specificity, positive predictive value (PPV), negative predictive value (NPV) were calculated after binarizing the continuous AI output using a predefined, vendor recommended threshold cut-off of 15, *which was the optimal threshold identified for adults.*”

We also changed the subheading in the results section as follows:

Page: 11, Line 9

“***Performance analysis based on the thresholds recommended for adults:***”

**Comment 11:**

The corrected analysis is interesting to highlight the imperfect reference standard (i.e., human radiologist). I think it highlights a limitation of the study that is not acknowledged in the discussion, that there was only one board-certified radiologist reviewing the X-rays compared to the 5 used in the adult study. This is evidenced by the fact that 6/7 corrected scores were actually when the AI was right but the labeller was wrong. I am not sure if re-calculating the diagnostic metrics for the corrected analysis makes sense as this is altering the definition of what the reference actually is. It may be sufficient to descriptively highlight these discrepant cases, and mention this lack of replication of radiological analysis as a limitation in the discussion.

**Response 11:**
We would like to thank the reviewer for this important observation. While one board-certified radiologist performed the reference readings, we would like to clarify that the original signed reports by pediatric radiologists were considered as second readings. Discrepant cases were subsequently reviewed and resolved through a consensus reading to establish the final reference standard for this study. We have acknowledged the limitation of not using a formal multi-reader consensus panel, in the Discussion section (see below)

Regarding the corrected analysis, we agree with the reviewer that recalculating diagnostic metrics based on retrospective case by case comparison could change the definition of the reference standard. We have therefore chosen not to include recalculated metrics and have thus omitted the paragraph “corrected performance analysis”. In keeping with this, we have removed the term “crude” performance analysis from the previous sections and have deleted the figure 2b. We now report these discrepancies descriptively in the Discussion.

Page: 17, Lines: 12-20

“*This could have been partially be off-set with a formal multi-reader consensus panel for ground truth reading to improve the replication of radiological analysis. Furthermore, during a focused review of cases to identify potential false positives, we identified a few cases such a discrete atelectasis or borderline cardiomegaly, which were flagged by the AI tool but not marked in the reference reads. While it is also important to note that AI may “overdiagnose” subtle findings that experienced radiologists would reasonably judge as clinically insignificant or not warranting formal reporting, this could suggest a potential role of AI tools in highlighting findings that could be overlooked in the clinical routine.”*

**Comment 12:**

“we calculated optimized cut-offs for each pathology and their performance metrics” – name the two pathology groups because I initially thought it was for each individual pathology (which is underpowered).

**Response 12:**

We would like to thank the reviewer for bringing this to our notice. We have changed the sentences as follows: (Page: 14, Lines: 21-23)

*“*The sub-group specific thresholds for pleuroparenchymal and mediastinal subgroups are shown in the **Fig 4** and for each pathology are shown in **S 1 Fig.***”*

**Comment 13:**

It would also be good to have an overall re-calculated thresholds for any abnormal vs normal for pediatric, as was done for adults.

**Response 13:**

We would like to thank the reviewer for this comment. We have now calculated the overall optimal threshold for children (Page:14, Lines: 17-21).

*“Overall, the optimal cut-off for the pediatric age group was found to be 44.5. This adjusted threshold yielded a similar AUC of 0.93 with an accuracy of 89.8% (95% CI: 87.7–91.6%), sensitivity of 80.0%, and specificity of 92.4%. Compared to the adult threshold, the pediatric-specific cut-off led to improvement in the specificity and the PPV, supporting a safer AI interpretation in children with a slight trade-off in the sensitivity at 80%.”*

We have updated the figure 4 accordingly (Page: 26)

**Comment 14:**

As previously mentioned, acknowledgement of a lack of replication of radiological interpretation in the reference needs to be acknowledged.

**Response 14:**

We thank the reviewer for highlighting this point. The following changes were made to the discussion. (Page: 17, Lines: 9-14)

“Finally, even though care was taken to generate high quality reference reads, minor variances in image interpretation such as missing/overdiagnosing slight pulmonary edema or atelectasis could not be avoided. *This could have been partially be off-set with a formal multi-reader consensus panel for ground truth reading to improve the replication of radiological analysis.”*

**Comment 15:**

Further limitations are that performance cannot be extrapolated to other respiratory conditions (e.g., tuberculosis CAD X-ray interpretation in adults vs in pediatrics cannot draw conclusions from this study). Could go along with the generalizability statement already made.
**Response 15:**
Thank you for the valid comment highlighting another important difference in disease manifestation in the pediatric population. We have added the following sentence to discussion (Page:16, Lines: 30-32):

*“Furthermore, the performance of the AI tool cannot be extrapolated to certain respiratory conditions like tuberculosis, where radiological manifestation of TB are different among the pediatric and adult populations”*

**Comment 16:**

Table 1: the pleuroparenchymal and mediastinal denominator should be 200. Would also be good to show the number who have both as a separate line .

**Response 16:**

We thank the reviewer for this point. We have updated the table 1 accordingly (Page: 17)

| ***Parameter*** | ***Total***  ***(n = 958)*** | ***Boys***  ***(n = 524)*** | ***Girls***  ***(n = 434)*** |
| --- | --- | --- | --- |
| ***Age (years)*** *** | *6.75 ± 3.6* | *6.83 ± 3.6* | *6.65 ± 3.6* |
| ***Projection (AP)*** | *500/958 (52.2 %)* | *274/524 (52.3%)* | *226/434 (52.1%)* |
| ***Relevant overall pathology*** | *200/958 (20.9 %)* | *96/524 (18.3%)* | *104 /434 (23.4%)* |
| *• Pleuroparenchymal pathology †* | *162/200 (81.0 %)* | *83/200 (41.5 %)* | *79/200 (39.5 %)* |
| *• Mediastinal pathology ‡* | *77/200 (38.5 %)* | *29/200 (14.5 %)* | *48/200 (24.0 %)* |
| *• Both mediastinal and pleuroparenchymal pathology* | *39/200 (19.5 %)* | *16/200 (8.0 %)* | *23/200 (11.5 %)* |

**Comment 17:**

Inconsistent N for the relevant pathology, pleuro, mediastinal across table 1, 2a and 2b. I expect the number in 1 to match 2a since 2b is the correct. However relevant overall in 1 is N=200 while in 2a is N=198, etc.

**Response 17:**

We would like to thank the reviewer for this and would like to apologize for this mistake. The values have been corrected (please refer to response 16).

**Comment 18:**

Figure 1: there is a lot of text, would be good to cut down. No definition of what (a) and (b) represent. Would be good to also show performance metrics for both adults and pediatrics so that it gives all the info in one place.

**Response 18:**

We thank the reviewer for this comment. The figure has been adjusted accordingly. The figure description was also changed to reflect the definition of (a) and (b). Since we have only evaluated the diagnostic performance in the pediatric population, we have only depicted the study methology in figure 1 and not included results. (Page: 6, Lines: 9-15)

***Fig 1: Brief summary highlighting the study methology****.* ***a)*** ***AI Tool Development and Pediatric Repurposing:*** *The AI tool was originally trained and validated using a large dataset of adult chest radiographs. For pediatric validation, the tool was retrospectively tested on 958 pediatric chest radiographs (CXR) from children aged 2–14 years.*

***b)*** ***Diagnostic Performance Analysis:*** *The AI tool’s diagnostic performance in children was assessed using vendor-recommended thresholds, stratified by age groups (2–6 and 7–14 years), and optimized pediatric-specific thresholds.*

**Comment 19:**

Figure 2: doesn’t add much to the manuscript, can be deleted.

**Response 19:**

We thank the reviewer for this feedback and have removed Figure 2. The numbering of other figures has been changed accordingly.

**Response to Reviewer 2**

We thank the reviewer for the critical evaluation of our work and are grateful for their very helpful comments and suggestions that we have incorporated in our revised manuscript as detailed below:

**Comment 1:**

Please revisit the keywords. My suggestion: Pediatric; Chest Radiograph; Artificial Intelligence

**Response 1:**

We have adapted the keywords as suggested: (Page: 2, Line: 3)

*“Pediatric; Chest Radiograph, Deep learning, Artificial Intelligence”*

**Comment 2:**

Rare --> largely unavailable; Any discordant findings --> All discordant findings
**Response 2:**

Corrected as suggested:

Page: 3, Lines: 2-3

“The number of commercially available artificial intelligence (AI) tools to support radiological workflows is constantly increasing, yet dedicated solutions for children are *largely unavailable*.”
(Page: 3, Line: 11)

*“All* discordant findings were reanalyzed in consensus.”

**Comment 3:**

The authors state: “The diagnostic performance of the AI-tool was validated using standard measures of accuracy using recommended and optimized thresholds to dichotomize the continuous AI output (0-100).” I think some readers might struggle with this and therefore ask the authors to elaborate on this.

**Response 3:**

We would like to thank the reviewer for this helpful suggestion. To improve clarity, we have made the following changes:

Abstract: Page: 3, lines: 12-15

*“The diagnostic performance of the AI-tool was validated using standard measures of accuracy. For this, the continuous AI output (ranging from 0-100) was binarized using vendor recommended thresholds recommended for adults and optimized thresholds identified for children.”*

Materials and methods: Page 8, Lines: 17-18

*“The software classifies the lesions as positive using a cut-off of 15%, which is the optimized for the adult population.”*

**Comment 4:**

The performance was high for relevant findings. Please elaborate what relevant means in this context.

**Response 4:**
We have added the following sentence to the abstract:

Page: 3, Lines: 15-18

*“Relevant findings were defined as consolidation, atelectasis, nodule, cardiomegaly, mediastinal widening due to mass, pleural effusion and pneumothorax. 200 radiographs [20.9%] demonstrated at least one relevant pathology.”*

**Comment 5:**

The readers may find the categorization according to the 7-year cut-off somewhat arbitrary. The authors may want to touch on this point, too.

**Response 5:**
The following sentence was added to the results:

(Page: 13, Lines: 26-29)

*“This age cut-off was chosen as it allowed for a balanced distribution of patients, allowing for statistically meaningful comparison between early childhood and school-aged children. Furthermore, in a previous study evaluating the same AI tool, 7 years was identified as the median age for correct diagnosis”*

**Comment 6:**

The authors have declared that no competing interests exist. With all due respect, this statement must be further justified given that the developer of the studied software tool supported the study and that one of the authors is affiliated with the company itself. I also notice that the authors report on page 17 that the developer financed two of the key authors through their institution. I warmly welcome full transparency. If possible, consider stating that although the developer funded the study and one of the authors is employed by the company, all authors had access to the data and the decision to publish was based on the findings, not by the request of the company.

**Response 6:**
We would like to thank the reviewer for their valuable feedback. One of the authors is employed by the company and has provided valuable technical support throughout the study. We would like to highlight that the company did not have any access to patient data and did not influence the decision to publish the results of our research. We have added the following statement to competing interests (Page: 20, lines: 4-6)

*“Competing Interests: Ambika Seth is affiliated with Lunit and provided technical support for the study and had no access to the patient data. The decision to publish was made independently of the company and was based solely on the scientific findings.”*

**Comment 7:**

Please also explicitly state the need for written informed consent was waived by the ethics committee and the study adheres to the national laws and regulations, if applicable.

**Response 7:**

We have stated this as follows: (Page: 6, Lines: 26-28)

*“This study was performed in line with the principles of the Declaration of Helsinki. Approval was granted by the Ethics Committee of University of Freiburg (22-1184-retro) and informed consent was waived.”*

**Comment 8:**

I note the authors use the terms chest radiograph and chest X-ray. Please stick to chest radiograph through and through.

**Response 8:**
We have changed the terms CXR and chest x-ray to chest radiograph throughout the manuscript.

**Comment 9:**

Although the introduction is well-written and reasoned, I think a more thorough review of existing tools for pediatric chest radiographs is very much needed. I therefore suggest well-conducted literature search and addition of a table summarizing the key results.

**Response 9:**
We would like to thank the reviewer for their valuable feedback. We have performed an updated search on the current status of commercially available AI tools for dedicated pediatric applications and have found that there is currently no tool for such use. Therefore, we cannot provide a tabular data on this.

We have added the following paragraphs to the introduction:

(Page: 4, Lines: 10-18)

*“Among the currently FDA-cleared commercially available AI solutions, there are, to date, no computer-aided detection products specifically authorized for pediatric use (7). This gap is attributable to several factors, including logistical challenges such as the limited availability of high-quality, open-access pediatric imaging datasets, and the scarcity of pediatric subspecialty radiologists required for accurate image annotation. Additional barriers include stricter regulatory requirements for pediatric research, smaller market potential compared to adult applications, and the inherent variability in clinical conditions and anatomical characteristics associated with the diverse age range within the pediatric population (8-10).”*

(Page 4, Lines: 26-30)

*“ In recent years, efforts have been made to adapt adult chest AI algorithms for pediatric use in order to accelerate the development of pediatric imaging AI. Various approaches have been explored, including the exclusion of children under two years of age and the omission of certain findings such as cardiomegaly to improve performance, as well as optimizing the operating thresholds of AI tools (11-13). “*

**Comment 10:**

The authors should describe the study as a single-center retrospective cohort study.

**Response 10:**

We have added this to the Materials and Methods section as follows:

Page: 6, Line: 3

“*In this single-center retrospective cohort study to independently externally validate a commercially available AI tool...*”

**Comment 11:**

Perhaps the authors would like to explain why they chose to include 1000 consecutive chest radiographs. What led them to think this is an appropriate size for the cohort, not for example 900 or 1100 chest radiographs?

**Response 11:**
We would like to thank the reviewer for this question.

The sample size of 1000 was a pragmatic trade-off to have a sufficiently large sample size to reasonably assess the diagnostic performance of the AI tool while accounting for the constraints of resources.

**Comment 12:**

I think it’s reasonable to omit the exclusion criteria 2 because the authors already stated they included radiographs of older children. Rather, please reason why the study was limited to children aged between 2-14 years.

**Response 12:**
We thank the reviewer for pointing this out. We have omitted the exclusion criteria 2 and have included the following sentence: (Page: 6, Lines: 18-22)

*“Children under the age of 2 years were not included in our study since they often show substantially differing anatomical features and a different spectrum of pathologies compared to older children. Since the AI tool is already approved for use in children older than 14 years, we limited the age group for our study from 2 – 14 years.”*

**Comment 13:**

Ethics statement: please make sure it’s concordant with the ethics statement as discussed earlier.

**Response 13:**

We confirm that the statement is concordant with previous statement.

**Comment 14:**

Fig. 3 --> Figure 3.

**Response 14:**
Corrected to suit the journal´s style as “Fig” throughout the manuscript.

**Comment 15:**

Please explain why all the available information, including the lab results, CT scans, and clinical history were taken into consideration when laying out the reference reads. After all, this information was not available to the software. While I of course support the use of all available information when reading the radiographs, one might argue that a truer performance assessment could have been achieved without considering other information.

**Response 15:**

We thank the reviewer for raising this important point. It is essential to clarify that the goal of our study was not to compare the performance of the AI tool against that of a radiologist. In such a head-to-head comparison, it would indeed be appropriate to blind the radiologist to additional clinical information to ensure a fair assessment.

However, in our study, the objective was to validate the AI tool’s performance in a real-world tertiary care setting. To ensure the highest possible accuracy in establishing reference standards or “ground truth”, we deliberately incorporated all available information, including clinical history, laboratory results, and CT imaging. This approach reflects the diagnostic process in clinical routine, where radiologists synthesize information from multiple sources to reach the most accurate conclusion.

**Comment 16:**

Were the chest radiographs caputed in supine, standing or sitting position?

**Response 16:**
As highlighted in table 1, 500 chest radiographs (52.2%) were obtained in AP projection and the remaining (458) chest radiographs were obtained in standing PA projection (Page 10).

**Comment 17:**

Rather than speaking of gender, please speak of sex. Men --> males, women --> females.

**Response 17:**

The necessary changes have been made (Page: 8, Line: 9)

**Comment 18:**

Was Lunit involved in the decision to publish the results?

**Response 18:**
We thank the reviewer for this important question. We would like to highlight that while Lunit was involved in providing technical assistance for the study, their team was not involved in the decision to publish the results.

**Comment 19:**

Could you please also touch on the training set used in the validation study. I think it would be very important to discuss the homogeneity/heterogeneity of the dataset particularly in relation to sex, age and ethnicity of the patients.

**Response 19:**
We would like to thank the reviewer for their question. We have contacted Lunit for this and here is their response.

The training and validation datasets were carefully curated to ensure a high degree of heterogeneity, both in terms of imaging conditions and patient demographics. We used a combination of public and private data sources from a wide range of geographic regions, including Europe, the United States, India, Vietnam, Israel, Mongolia, and South Korea. This helped ensure representation across diverse age groups, sexes, and ethnicities, enhancing the generalizability of the model. Additionally, images were obtained from more than 24 device models from vendors such as Philips Medical Systems, GE Healthcare, FUJIFILM Corporation, Siemens, Canon, Carestream Health, Agfa Healthcare, and others, capturing variability in hardware, image quality, and clinical settings. This broad range of sources contributes to the robustness and performance consistency of the model across different patient populations and imaging environments.

We have added the following line to our manuscript (Page:8, Lines: 4-7)

*“For training of the AI, 262,445 adult frontal chest radiographs (146,679 normal and 115,766 abnormal) were used from various vendors and with a wide range of acquisition parameters and across a wide range of geographic regions.”*

**Comment 20:**

Statistical Analysis: I wish to applaud for the well-written chapter. Normally this section is overlooked. I find it easy to immerse into the Results section after reading this chapter. However, I ask the authors to elaborate on the age categorization.

**Response 20:**

Please refer to response 5.

**Comment 21:**

All in all, this section is well written and nicely backed by the tables. However, as the tables should stand alone, I think the authors could think of further strengthening the tables by informing the reader where the numbers come from. For example, it should be clear what is being used as the gold standard and why the number of relevant overall pathologies differ between the tables.

**Response 21:**

We have added the following sentence in the table description:

Page: 12 , Lines: 1-2

*“For the calculation of the* *diagnostic performance of the AI tool, dedicated reference reads generated for this study were used as a gold standard.”*

**Comment 22:**

Please also report the results for girls/boys separately in Table 1.

**Response 22:**

We have edited the table 1 accordingly. Kindly refer to response 16 to Reviewer 1.

**Comment 23:**

I’d also encourage the authors to report the ethnicities of the patients.

**Response 23:**

We appreciate the reviewer’s thoughtful comment regarding the categorization of ethnicity in our study. We would like to highlight that the concept and classification of “ethnicity” are primarily shaped by U.S.-based frameworks and may not accurately reflect the ethnic diversity or classifications relevant to the German population, where the population is relatively homogenous. The vast majority of the 958 pediatric patients included in the study were “white”, with approximately 35 patients estimated to belong to other backgrounds. We have now highlighted this as a limitation of our study in our revised manuscript (please refer to response 24).

**Comment 24:**

I’d like the authors to also discuss whether the results would translate to all demographic subgroups of children. Particularly, does ethnicity have an effect?

**Response 24:**

Thank you for this important question. We acknowledge that ethnicity and other factors that may be related to it, such as anatomical variations, socioeconomic status, and access to care may influence disease presentation and diagnostic performance of AI tools, as highlighted in certain studies reporting underperformance of AI models in certain ethnicities (for example *Coarse Race and Ethnicity Labels Mask Granular Underdiagnosis Disparities in Deep Learning Models for Chest Radiograph Diagnosis*, P. Bachina, et. al. Radiology 2023). However, due to the small sample size of these subgroups, we were unable to perform stratified analyses to evaluate performance differences by ethnicity.

We have now added a paragraph to the Discussion section (Page: 17, Lines: 5-9) acknowledging this limitation and emphasizing the need for further research in more ethnically diverse pediatric populations to assess the generalizability of our findings.

*“Our study was conducted in a predominantly European pediatric population, reflecting the demographic composition of the study region. Future studies involving more ethnically diverse cohorts are warranted to assess the generalizability and fairness of AI applications in pediatric imaging, and to account for potential algorithmic biases related to ethnicity”*

**Comment 25:**

Table 2b:

“a simplified manner to avoid bias due to low number of cases.” please rewrite for clarity.

**Response 25:**
We would like to thank the reviewer for this comment. Figure 2b was deleted as per the comments of Reviewer 1.

**Comment 26:**

Fig 1

Please also indicate the study setting: this is a single-center retrospective cohort study.

**Response 26:**

We have edited the figure and now included a sentence on the study setting.

**Comment 27:**

Fig 1

It said in the results 200 (20.9%) of the chest radiographs had at least one pathology. Figure 1 seems to suggest there were 198 chest radiographs with abnormal findings.

**Response 27:**
We would like to thank the reviewers for bringing this to our notice and apologize for this oversight. We have corrected the figure accordingly.

**Comment 28:**

Of note, it seems the fonts are widely inconsistent between the Figures

**Response 28:**

We thank the author for highlighting this. We have replaced the fonts in the figures.

**Comment 29:**

Please name the Figure descriptively keeping in mind that the Figure and the description should be able to “stand alone”, i.e., be clear without reading the text.

**Response 29:**
We thank the reviewer for their comment. We have added the following description to figure 1:

Page: 6, Lines: 9-15

**Fig 1: Brief summary highlighting the study methology**. **a)** **AI Tool Development and Pediatric Repurposing:** The AI tool was originally trained and validated using a large dataset of adult chest radiographs. For pediatric validation, the tool was retrospectively tested on 958 pediatric chest radiographs (CXR) from children aged 2–14 years.

**b)** **Diagnostic Performance Analysis:** The AI tool’s diagnostic performance in children was assessed using vendor-recommended thresholds, stratified by age groups (2–6 and 7–14 years), and optimized pediatric-specific thresholds.

**Comment 30:**

If the name of the title refers to the CONSORT reporting system, the word should be fully capitalized. Please correct the inconsistent spacings.

**Response 30:**
As per the recommendations of Reviewer 1, figure 2 has been deleted.

**Comment 31:**

Figure 3

There seems to be some kind of an annotation in the upper right corner of the A part. Was this intentional?

**Response 31:**

Thank you for this question. The box in the upper right corner in figure 3 (now figure 2) A depicts the annotation tool of our in-house image-processing platform NORA. For the sake of clarity, we have included the following sentence in the manuscript: (Page: 7, Lines: 27-28)

*“The box in the upper right corner shows the annotation tool of the image-processing platform NORA.”*

**Comment 32:**

Figure 5:

Not sure if the figure is needed. I would not oppose its inclusion, however, I feel it does not really add too much information. Should the authors want to keep it, I’d ask them to avoid abbreviations sens, PPV, NOV and spec.

**Response 32:**

We thank the reviewer for their valuable input. We have decided to include the figure, as it helps to visualize the performance using optimized thresholds for children compared the thresholds recommended for adults. Due to space constraints, we chose to include abbreviations and have now added their explanations as follows (Page 14, Lines: 31-32):

*(sens = sensitivity, spec = specificity, PPV = positive predictive value, NPV = negative predictive value)*
